# Highly parallelized human embryonic stem cell differentiation to cardiac mesoderm in nanoliter chambers on a microfluidic chip

**DOI:** 10.1007/s10544-021-00556-1

**Published:** 2021-05-31

**Authors:** Anke R. Vollertsen, Simone A. ten Den, Verena Schwach, Albert van den Berg, Robert Passier, Andries D. van der Meer, Mathieu Odijk

**Affiliations:** 1 BIOS Lab On a Chip Group, MESA+ Institute for Nanotechnology, Max Planck – University of Twente Center for Complex Fluid Dynamics, University of Twente, Enschede, The Netherlands; 2 Applied Stem Cell Technologies, TechMed Centre, University of Twente, Enschede, The Netherlands

**Keywords:** Microfluidic large-scale integration, Human pluripotent stem cells, Cardiac mesoderm, Parallelization, Discontinuous perfusion

## Abstract

Human stem cell-derived cells and tissues hold considerable potential for applications in regenerative medicine, disease modeling and drug discovery. The generation, culture and differentiation of stem cells in low-volume, automated and parallelized microfluidic chips hold great promise to accelerate the research in this domain. Here, we show that we can differentiate human embryonic stem cells (hESCs) to early cardiac mesodermal cells in microfluidic chambers that have a volume of only 30 nanoliters, using discontinuous medium perfusion. 64 of these chambers were parallelized on a chip which contained integrated valves to spatiotemporally isolate the chambers and automate cell culture medium exchanges. To confirm cell pluripotency, we tracked hESC proliferation and immunostained the cells for pluripotency markers SOX2 and OCT3/4. During differentiation, we investigated the effect of different medium perfusion frequencies on cell reorganization and the expression of the early cardiac mesoderm reporter MESP1^mCherry^ by live-cell imaging. Our study demonstrates that microfluidic technology can be used to automatically culture, differentiate and study hESC in very low-volume culture chambers even without continuous medium perfusion. This result is an important step towards further automation and parallelization in stem cell technology.

**Supplementary information:**

The online version contains supplementary material available at 10.1007/s10544-021-00556-1.

## Introduction

Stem cell technology advances the generation and controlled differentiation of stem cells, including human adult stem cells, human embryonic stem cells (hESCs) and human induced pluripotent stem cells (hiPSCs). Stem cells have enabled new strategies for use in fields like regenerative medicine, disease modeling and drug discovery. In particular, stem cell technology enables the controlled generation and use of cell types, such as cardiomyocytes, which are difficult to obtain as human primary cells. Importantly, the use of human adult stem cells and hiPSC has paved the way for creating patient-specific *in vitro* tissues, organoids (Yin et al. 2016; Dutta et al. 2017; Drost and Clevers 2018) and organs-on-chips (Huh et al. 2011; Van Der Meer and Van Den Berg 2012; Bhatia and Ingber 2014) that can be used in personalized drug development and precision medicine (Luni et al. 2014; Clevers 2016; Van Den Berg et al. 2019). An important aspect of engineering stem cell differentiation is understanding and mapping the involved signaling pathways by being able to tightly control the cells’ microenvironments.

Directing stem cell fate of human pluripotent stem cells (hPSCs) is essentially achieved by emulating embryogenesis and thus requires creating a highly dynamic and spatially heterogenous microenvironment. Consequently, reliably and reproducibly generating cell lineages with a high degree of cell commitment presents an enormous challenge. Significant progress has been made to overcome this challenge by systematically mapping ‘decision points’ in the differentiation pathways (Loh et al. 2016). Yet this approach, performed in standard well plate culture, incurs a high cost in either manual labor or expensive automation equipment as well as large amounts of costly reagents. Moreover, future testing of non-chemical environmental stimuli, such as neighboring tissues, is limited. These limitations can be overcome in microfluidic systems, and therefore developing stem cell differentiation protocols in microfluidic devices is an extremely promising domain (Kshitiz et al. 2011; Park et al. 2015; Zhang et al. 2017).

Research in microfluidic stem cell differentiation covers a wide variety of aspects, including hydrogel encapsulation (Figallo et al. 2007; Alessandri et al. 2016; Henke et al. 2016), substrate surface modification (Abhyankar and Beebe 2007; Hesari et al. 2016; Kamei et al. 2017), factorial stimuli screening (Titmarsh et al. 2017; Chadly et al. 2019) and spatiotemporal stimuli screening (Gómez-Sjöberg et al. 2007; Wu et al. 2016; Zhang et al. 2019). Several studies underline the importance of maintaining tight control over the cells’ microenvironment and demonstrate that this goes beyond culturing with fully-defined medium(Przybyla and Voldman 2012b; Giobbe et al. 2015; Luni et al. 2016). Cells alter their direct environment by producing autocrine signaling factors which influence their development (Przybyla and Voldman 2012a; Gagliano et al. 2016; Guild et al. 2016). Refreshing the culture medium removes these signaling factors. Consequently, the medium perfusion rate changes the overall mean level of cell-secreted factors, making it an important parameter in microfluidic stem cell culture (Titmarsh et al. 2011; Yoshimitsu et al. 2014; Abdolvand et al. 2019; Fattahi et al. 2020). The optimal perfusion rate, in turn, depends on the volume of the cell culture space. Given smaller medium volumes per cell, cell-secreted factors can accumulate faster while exogenous factors are depleted more quickly (Giobbe et al. 2015). In particular, the effect of confined medium volumes on stem cell differentiation was shown by G. Giobbe et al., who used 5 µL channels to enhance hESC and hiPSC differentiation towards different germ layers by varying discontinuous medium perfusion frequencies (Giobbe et al. 2015). Recently, the same work group used similar microfluidic culture systems to reprogram human somatic cells to hiPSC at a significantly higher efficiency than in well plates (Luni et al. 2016; Gagliano et al. 2019). They attributed the increased efficiency to the faster accumulation of endogenous factors in smaller volumes.

Further reduction in microfluidic culture volume for hPSC differentiation has great potential. Most importantly, it allows even tighter control over the cells’ microenvironments. Smaller volumes for faster endogenous factor accumulation could allow higher medium perfusion frequencies, which can be used to mimic a highly dynamic environment. Furthermore, decreasing the channel footprint increases the amount of different conditions which can be potentially screened in the same area. Finally, the amount of medium and differentiation factors required per screened condition are reduced even further. Although there have been some studies on optimizing perfusion rates for hPSC differentiation in sub-microliter volumes, these have mainly focused on continuous perfusion strategies in connected microwells (Lee et al. 2011; Titmarsh et al. 2011). However, interconnected culturing volumes do not prevent cross-diffusion of cell-secreted factors and are limited in versatility for screening different perfusion rates.

Screening small chambers with different medium perfusion frequencies requires that they can be addressed with spatial and temporal independence. For passive microfluidic chips, more external equipment (e.g. syringe pumps) is needed to run independent protocols for each culturing space, limiting overall throughput. In microfluidic systems with integrated active components (such as valves (Unger et al. 2000; Melin and Quake 2007)), it is possible to achieve tens to hundreds of parallelized, spatiotemporally isolated conditions (Thorsen et al. 2002; Gómez-Sjöberg et al. 2007; Sikorski et al. 2015; Wu et al. 2016; Zhang et al. 2019). Microfluidic large-scale integration (mLSI) chips (Thorsen et al. 2002) contain hundreds of integrated valves which can automate up to millions of pipetting steps (Zhang et al. 2019). Long-term (weeks) cell culturing experiments on such mLSI platforms have been performed in chambers with volumes in the tens of nanoliters range. Although these systems have been used to culture hESCs (Kamei et al. 2009; Sikorski et al. 2015), human mesenchymal stem cells (Gómez-Sjöberg et al. 2007; Wu et al. 2016) and murine neural stem cells (Zhang et al. 2019), they have yet to be used to study the effect of different perfusion frequencies on the directed differentiation of hPSCs.

Previously, we reported a standardized and scalable system featuring an mLSI chip with 64 parallelized and independently addressable chambers. Each chamber has a volume of 30 nL in which we cultured human umbilical vein endothelial cells for several days (Vollertsen et al. 2020). Finding optimal perfusion frequencies to enhance the differentiation yield of cells is expected to be a complex problem because the optimal frequency depends on the stage of differentiation and associated medium, requiring multiple parallel screens for each stage. In this work, we focus on validating our chip for culturing and differentiating hESCs to cardiac mesodermal cells as the first step towards addressing this complex problem. We show that the medium perfusion frequencies affect the cells’ self-organization during the 3-day differentiation as well as their expression of the early cardiac mesoderm reporter MESP1 by live-cell imaging. In addition, we show that we obtain a twice as high yield of MESP1-expressing cells by culturing cells in the chambers as compared to the well plate.

## Results and discussion

### 64-chamber chip

**Fig. 1:**
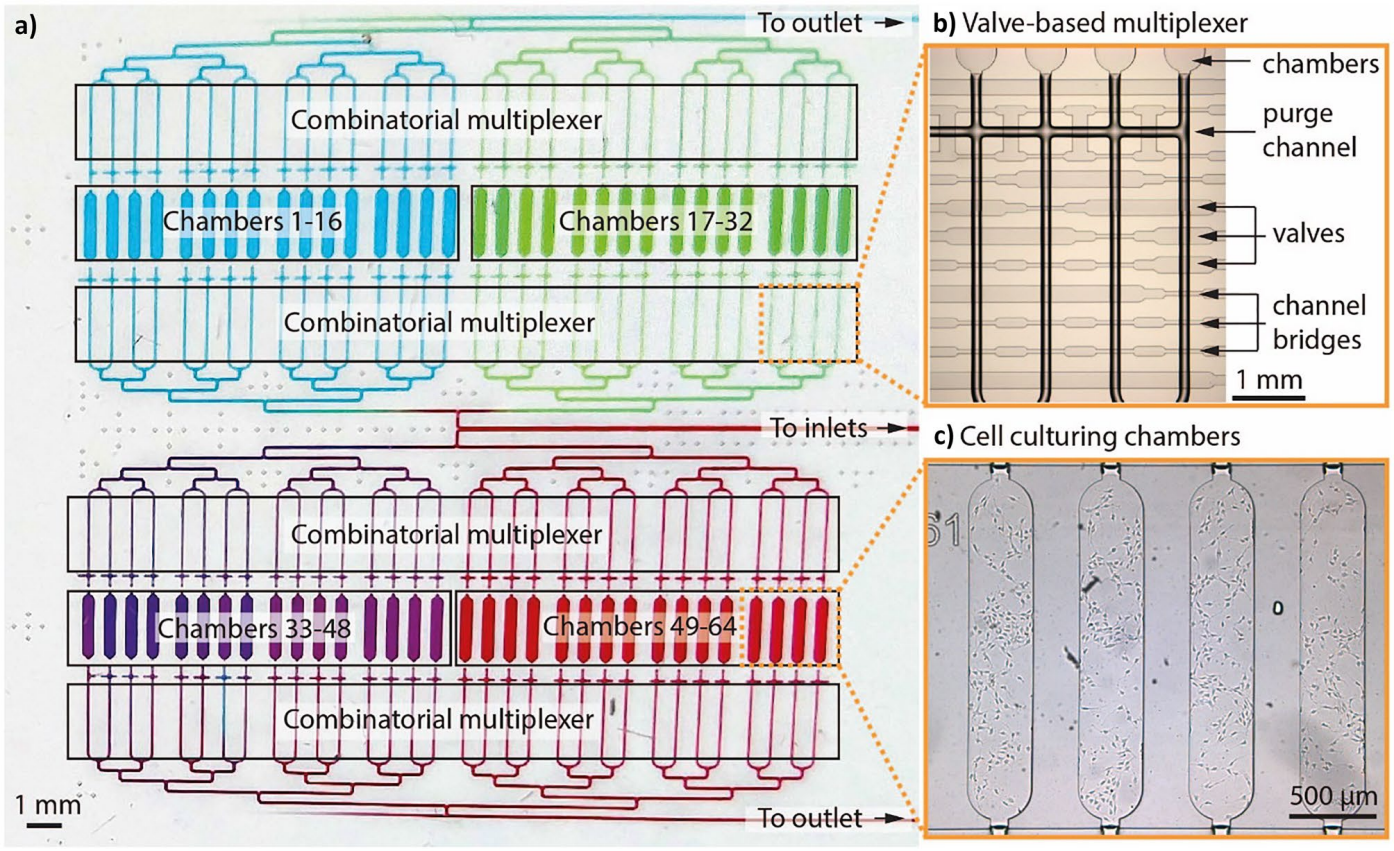
Microfluidic chip used for hESC differentiation. (**a**) Photograph of the chip where each quarter is filled with a different color of food dye. (**b**) Brightfield micrograph of the valve multiplexer used to independently address the chambers. The light channels are the control channels which orthogonally cross the flow channels (dark channels). Wide cross sections form valves which close off the flow channels when the control channels are pressurized. Thin cross sections (bridges) are insufficient to close off the overlaying flow channels

The microfluidic chip used in these experiments was previously reported by us (Vollertsen et al. 2020). In short, the PDMS chip features 64 independently addressable chambers for cell culturing with a volume of 30 nL each. Figure 1a shows a photograph of the chip where chambers in each quarter are filled with a different color of food dye. The chambers can be addressed using a valve-based combinatorial multiplexer (Hua et al. 2006) as shown in Fig. 1b. The multiplexer is mirrored on the other side of the chambers to prevent a net flow through non-addressed chambers where the multiplexer valves may peristaltically push or pull liquid as a side effect of other chambers being addressed. A purge channel (Fig. 1b) located between the multiplexer and the chambers connects the channels so that these can be purged of their content without contaminating the chambers. This aspect is important for both the surface modification of the chip and the removal of unwanted cells in the channels after cell seeding in the chambers. Figure 1c shows a brightfield image of four of the chambers containing hESC after 24 h of automated cell culturing.

The low volumes of the chip are an essential aspect for reducing the cost of screening for optimal conditions for stem cell differentiation, as the main cost factor for such experiments is the cell culture medium and its components. Clearly, our experimental microfluidic platform currently costs a lot more to fabricate than commercial well plates, but the cost of manufacturing could be reduced dramatically by automating fabrication.

Another aspect to consider in future chip fabrication is the use of PDMS. This material has been widely discussed and reviewed due to both its absorption of small hydrophobic molecules and potential release of uncrosslinked polymers (Halldorsson et al. 2015; van Meer et al. 2017). For full certainty that these material properties don’t influence the stem cell differentiation by changing the ratio of externally-added to cell-secreted factors it would be best to use devices made from polymers such as polystyrene or PMMA. The adverse effects of PDMS should be noted in particular for testing differentiation pathways towards certain cell types. For example, human neuronal stem cell culture has been shown to result in widespread cell death in PDMS devices (Kajtez et al. 2020). However, for stem cell differentiation towards cardiac mesodermal cells (or even full cardiomyocytes), we do not expect the PDMS absorption of molecules to greatly hinder the differentiation. It has been previously shown that cardiomyocytes can be derived from stem cells in PDMS devices (Giobbe et al. 2015). Furthermore, we have also successfully derived cardiomyocytes from stem cells on PDMS (though admittedly a different brand) in well plates (data not shown).

### HESC population change depending on different medium exchange intervals

The effect of culturing hESCs in the 30 nL chambers with different medium exchange rates was investigated by monitoring the cells’ proliferation rates. For easier representation, the exchange frequencies are henceforth expressed as intervals between medium exchanges (e.g. every 5 h instead of 4.8 d^−1^). Four different medium exchange intervals (2 h, 3 h, 5 h, and 8 h) with 14–16 replicates per interval were tested on a single chip. On average, there was less medium per cell per hour in the chambers for all conditions than in the control 6-well plate (Table 1). These time intervals were chosen to allow more time for endogenous factors produced by the cells to accumulate in the chambers between medium refreshments, since these factors are removed along with every medium refreshment.

**Table 1:** Average cell medium per hour and cell for hESC culture and differentiation in a 6-well plate and in chambers with 1 h, 2 h, 3 h, 5 h, and 8 h exchange intervals

hESC culture: Day -1 to 0					
Condition	6-well plate (2 mL for 24 h)	2 h	3 h	5 h	8 h
Assumed cells per well/ chamber	125 000	100	100	100	100
Medium per hour per cell [nL h^−1^ cell^−1^]	0.67	0.15	0.10	0.06	0.04

Differentiation to cardiac mesoderm: Day 0 to 3					
Condition	6-well plate (3 mL for 72 h)	1 h	2 h	3 h	5 h
Assumed cells per well/ chamber	250 000	150	150	150	150
Medium per cell per hour [nL h^−1^ cell^−1^]	0.17	0.20	0.10	0.07	0.04

**Fig. 2:**
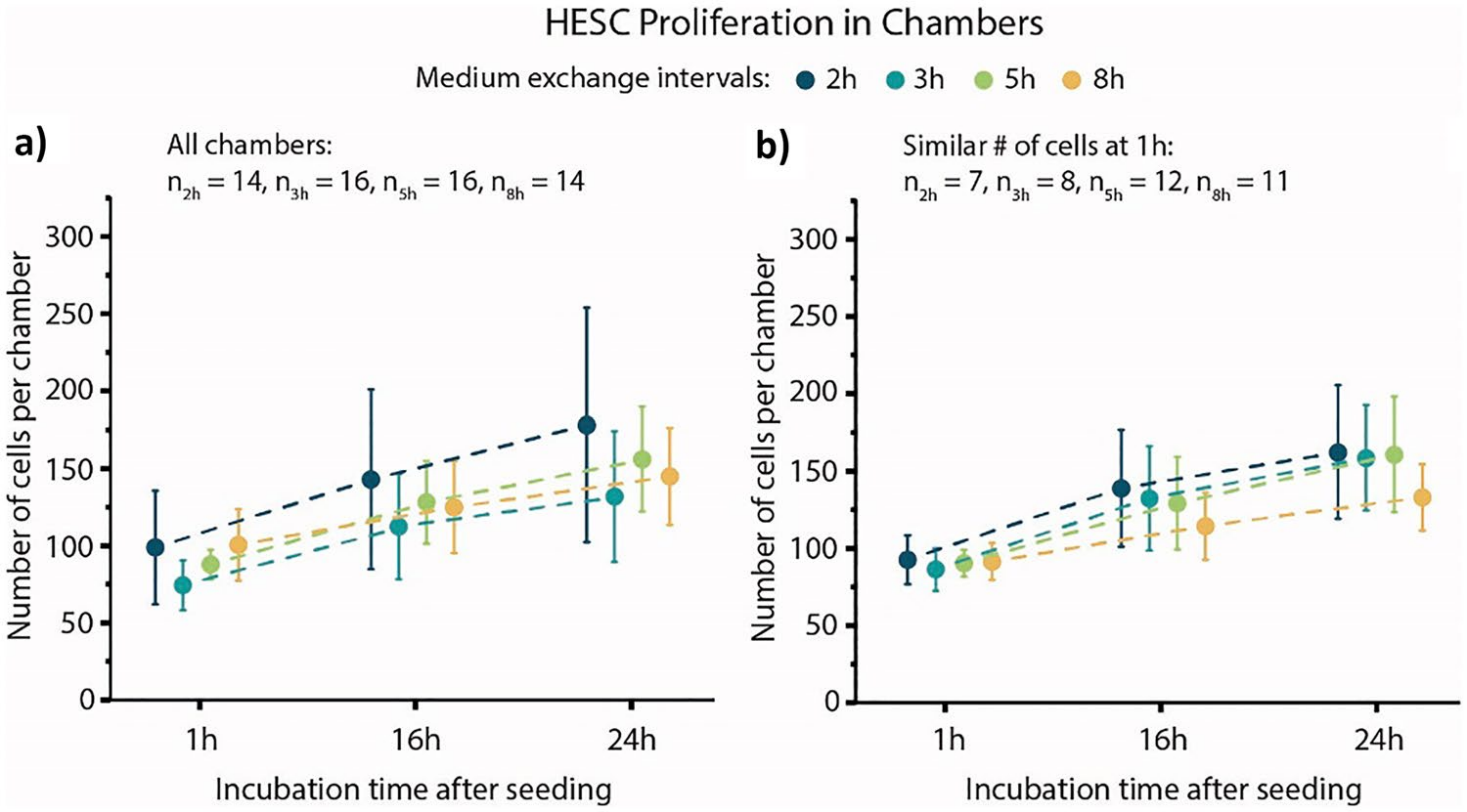
HESC proliferation in microfluidic chambers. (**a**) Means and standard deviations for all chambers at 1 h, 16 h, and 24 h after cell seeding. The number of chambers per condition (n) is between 14 and 16 chambers. (**b**) Means and standard deviations for a subset of chambers with similar cell populations at 1 h (between 69 and 108 cells per chamber). n ranges from 7 to 12 chamber per condition

Figure 2a shows the mean number of cells per chamber for each medium exchange interval at 1 h, 16 h, and 24 h after seeding. After seeding, most chambers contained between 70 and 120 cells. The cell counts at 16 h and 24 h after seeding show cell proliferation for all four intervals. However, it also becomes apparent that the average cell number per chamber at the beginning of the experiment (1 h) differs by up to 27% between the four conditions. For example, the 8 h chambers have an average of 101 cells per chamber, while the 3 h chambers only have an average of 74 cells per chamber. As a result, there is more medium available per cell (in between medium exchanges) in some of the conditions, which may also have an effect on the proliferation. Therefore, a subset of the chambers is shown in Fig. 2b, where all chambers contained on average approximately 90 cells at 1 h after seeding. The subset contains between *n* = 7 (2 h) and *n* = 12 (5 h) chambers per condition. A repeated measures ANOVA (analysis of variance) showed no statistically significant difference between the conditions. In addition, a second experiment with 1h, 2h, 3h, and 5h medium exchange intervals was performed, which likewise showed no statistically significant difference between conditions (Suppl. Information 1).

The results show that the hESCs remain highly proliferative for at least the first 24 h even with only tens of nanoliters of medium and long exchange intervals (5-8 h). Furthermore, they show that frequent medium exchanges at low shear stress τ (τ < 0.13 Pa) directly after cell seeding do not result in decreased cell proliferation. This suggests that the common practice of leaving hESCs to attach overnight in microfluidic devices without medium refreshment may not be necessary.

### Pluripotency staining of hESCs in chambers

**Fig. 3:**
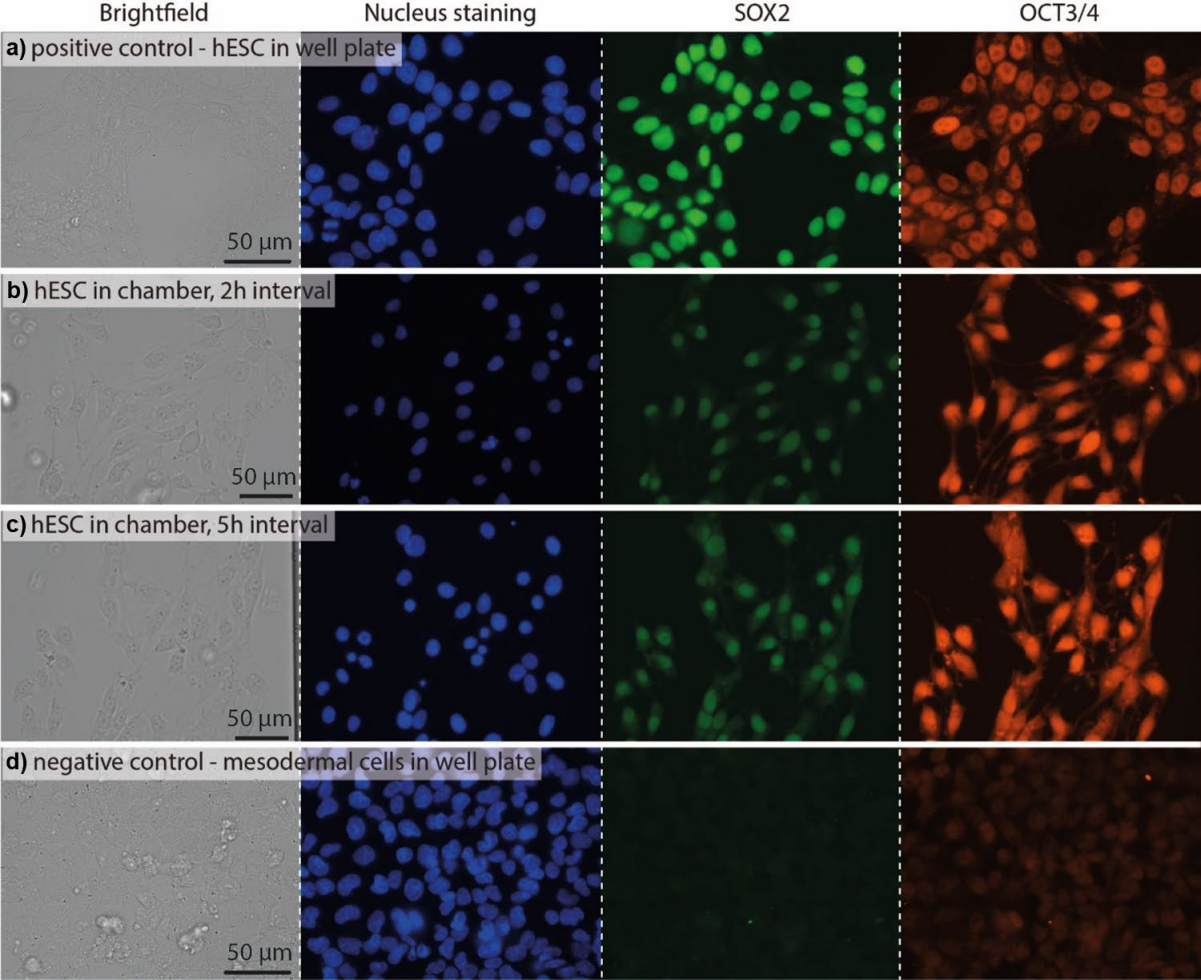
Brightfield, fluorescent nuclei staining and immunofluorescence (SOX2 in pseudo-green and OCT3/4 in pseudo-orange) images of hESCs after 24 h of culture in (**a**) a well plate, (**b**) in chambers with a 2 h medium exchange interval, and (**c**) in chambers with a 5 h medium exchange interval. d) For comparison the same stainings were performed on cardiac mesodermal cells in well plates derived from the same hESC line on day 3 of differentiation. All images were taken with the same microscope (EVOS FL2 auto). The well plate images were taken with the same objective and intensity/exposure settings. The chamber images were both taken with an objective with a higher working distance than the objective used for the well plates. Both chamber images were taken with the same objective and intensity/exposure settings. In the well plates DAPI was used to stain the nucleus whereas NucBlue was used in the chambers

We confirmed that the hESCs remained pluripotent while they were proliferating in the microfluidic chip. HESC pluripotency on day 0 (24 h after seeding) in the microchambers was confirmed by immunostaining for the pluripotency markers SOX2 and OCT3/4. On day -1, the cells were seeded at approximately 100–150 cells per chamber and Essential 8 (E8) medium was exchanged every 2 h and every 5 h for half of the chambers, respectively. On day 0, the cells were fixed and stained. As a positive control, hESCs cultured in well plates were also immunostained. The brightfield and fluorescence images are shown in Fig. 3 (the fluorescent images are pseudo-colored whereby the nucleus staining is shown in blue, SOX2 immunostaining in green and OCT3/4 immunostaining in orange). Both the 2 h and 5 h conditions show the presence of SOX2 and OCT3/4 markers, indicating pluripotency. For comparison, cells which had been differentiated towards the cardiac mesoderm (for 3 days) in well plates were also fixed and stained for SOX2 and OCT3/4. As expected, the cardiac mesodermal cells no longer show SOX2 expression and show significantly lower OCT3/4 expression compared to hESCs on day 0. Although OCT3/4 is a marker for pluripotency, OCT4 also regulates the switch from SOX2 to SOX17 expression which directs the cell towards a cardiac fate (Zeineddine et al. 2006; Stefanovic et al. 2009). Therefore, OCT4 is present in the cells for longer than SOX2 in this differentiation process which could explain the residual expression observed in the cardiac mesodermal cells.

### Cell re-organization during differentiation to cardiac mesodermal cells

We performed an automated differentiation experiment in which we differentiated hESCs into early cardiac mesoderm. Differentiation medium containing Activin A, BMP-4, and CHIR (ABC medium) replaced the E8 medium in the chambers on day 0. Medium exchange intervals were set to 1 h, 2 h, 3 h and 5 h. Prior to the start of differentiation, the same medium exchange interval (2 h) had been used in all chambers after seeding. Each chamber contained between about 100 and 150 cells on day 0. Table 1 shows the average volume of medium per cell per hour assuming approximately 100 cells per chamber. On average, the cells in the 1 h and 2 h chambers come into contact with roughly the same amount of medium as the cells in the well plate in the three days of differentiation.

**Fig. 4:**
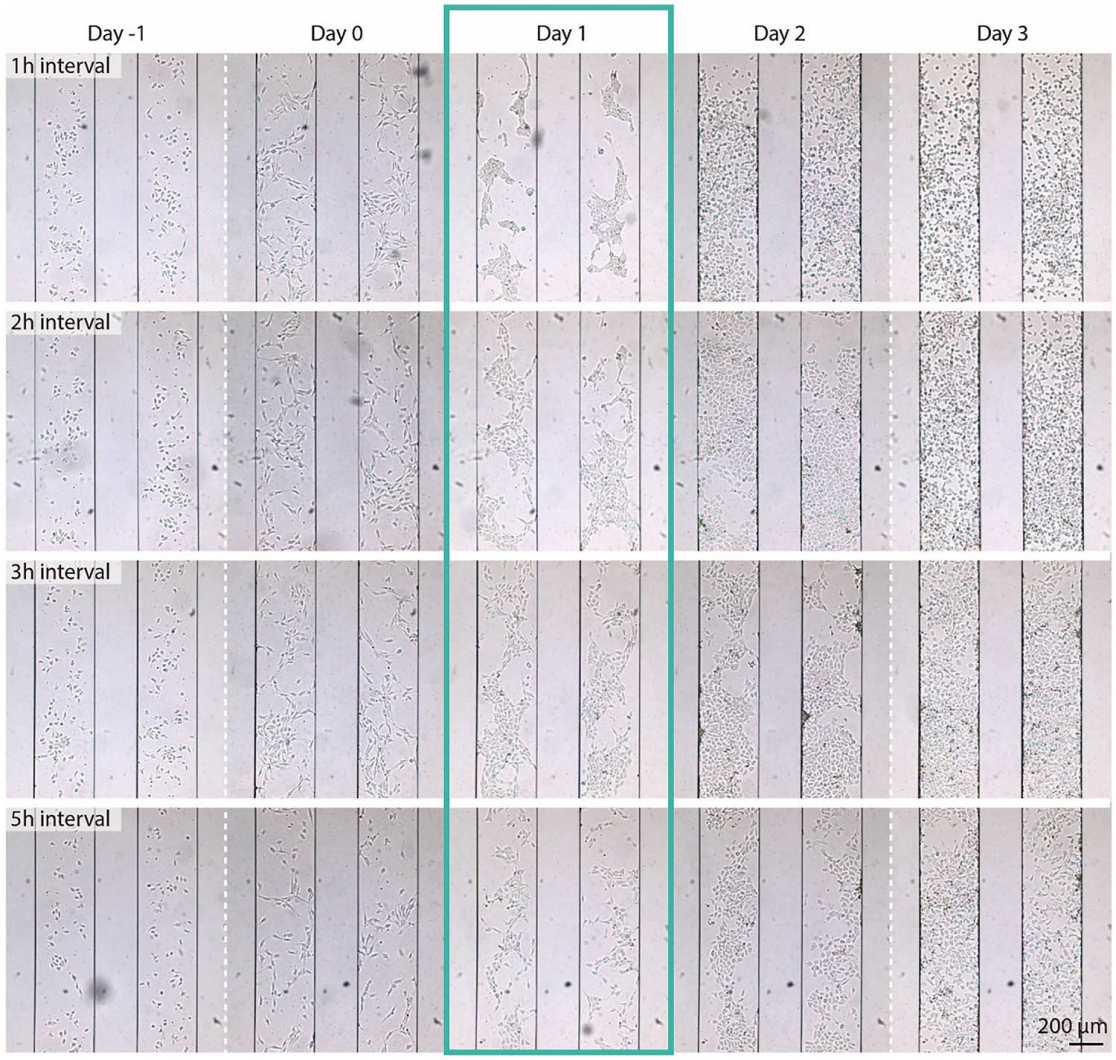
hESC morphology and re-organization after seeding (day -1) and during differentiation from day 0 to day 3. The shorter the medium exchange interval, the more compact the cell groups are on day 1. For the 1 h intervals the cells disperse as single cells from the aggregates on day 2. For the other conditions, this is observed on day 3. In all cases except for the 1 h chambers, the cells show clear proliferation from day 2 to day 3, resulting in full chambers on day 3

Figure 4 shows brightfield images of two of the chambers for each condition on each day. On day 1, a clear difference is seen between the 1 h interval condition and the other three conditions. In the 1 h chambers, the cells are no longer spread evenly throughout the chambers, but instead have condensed in groups to form aggregates. This aggregate formation is also observed in well plates (Supplementary Fig. 2a). In comparison, the cells treated with 2 h and 3 h exchange intervals formed less compact groups with less defined edges. The Supplementary Video shows a time lapse of the cells compacting in the 2 h chambers. In contrast to the 1 h chambers, the cells in these chambers are still individually distinguishable. For the 5 h chambers a continuation of this trend can be observed, as there is only some grouping of cells and several cells remain as single cells. On day 2, the cell aggregates in the 1 h chambers have dispersed and spread throughout the chamber. These cells have a different morphology than the hESCs on day 0. Whereas the hESCs on day 0 are elongated, these day 2 cells are round and circular. Interestingly, the 2 h chambers on day 2 did not form aggregates even though by this time the same amount of ABC medium has flowed through the cells as was flowed through the 1 h chambers by day 1. A possible explanation for this could be the cell proliferation from day 1 to day 2 resulting in less available differentiation factors per cell. In general, the cells in the 2 h, 3 h and 5 h chambers on day 2 show a monolayer with cobblestone-like morphology and only occasional small aggregates at chamber sides (as seen in Fig. 4 in the 3 h chambers). All conditions show high cell proliferation. On day 3 the chambers in all conditions are filled with cells which have dispersed to cover most of the chamber floor. In the 1 h chambers wider gaps between cells are visible, indicating some cell death and a decrease in proliferation. The cell morphology and distribution in the 2 h chambers are comparable to those in the 1 h chambers on day 2. The cells in both the 3 h and 5 h chambers also show high proliferation, resulting in (nearly) confluent chambers. Here the cell morphology has also changed to resemble that of the cells in the 2 h chambers despite the lower total amounts of ABC medium which have passed through the chambers.

In an earlier experiment, differentiation was started when there were about 300–400 cells per chamber. In that case the cells in the 1 h chambers also formed condensed aggregates on day 1. However, these were still present on day 2 and the cells only started to disperse between days 2 and 3. For all medium exchange intervals, the cells greatly overpopulated the chambers by day 3. These images are shown in Supplementary Fig. 3. These results show that a lower seeding density is essential for obtaining a cell monolayer on day 3 when using these growth factor concentrations (20 ng/mL Activin A, 20 ng/mL BMP-4, and 1.5 µM CHIR).

For the experiments in this section, live-cell imaging was the method of choice to be able to show the same cells in the chambers over the course of differentiation. Future studies, for example for more detailed cell morphological changes, would also benefit from immunostaining at different time points.

### MESP1 expression on day 3

Differentiation into cardiac mesodermal cells was confirmed by fluorescence imaging of the MESP1^mCherry^-hESC reporter line (Den Hartogh et al. 2015). Previously, we have shown that MESP1 is transiently activated, peaking at day 3, during cardiac differentiation of hESCs (Den Hartogh et al. 2015). Here we confirm that the cells cultured in the chip also express MESP1^mCherry^ on day 3 by performing flow cytometry and comparing the results to negative controls (hESCs) and positive controls (day 3 cardiac mesodermal cells cultured in a well plate). In order to obtain a sufficient amount of cells, the cells from all chambers were extracted and pooled. The flow cytometry results (Supplementary Fig. 4) show that the percentage of MESP1-expressing cells in the chambers was about twice as high as in the well plates (Table 2). However, since the chip was designed to test several different conditions in parallel, we decided to use fluorescence microscopy for further experiments. Although the method is not suited to determining absolute percentages of MESP1-expressing cells within a population, it is well-suited to making relative comparisons between several different conditions. Moreover, it has the advantage that each chamber can be compared.

**Table 2:** Percentages of MESP1^mCherry^ expressing cells determined by flow cytometry

Condition	Undifferentiated	Well plate culture	Chamber culture
Percentage of MESP1^mCherry^ expressing cells	0.7%	11.4%	24.5%

**Fig. 5:**
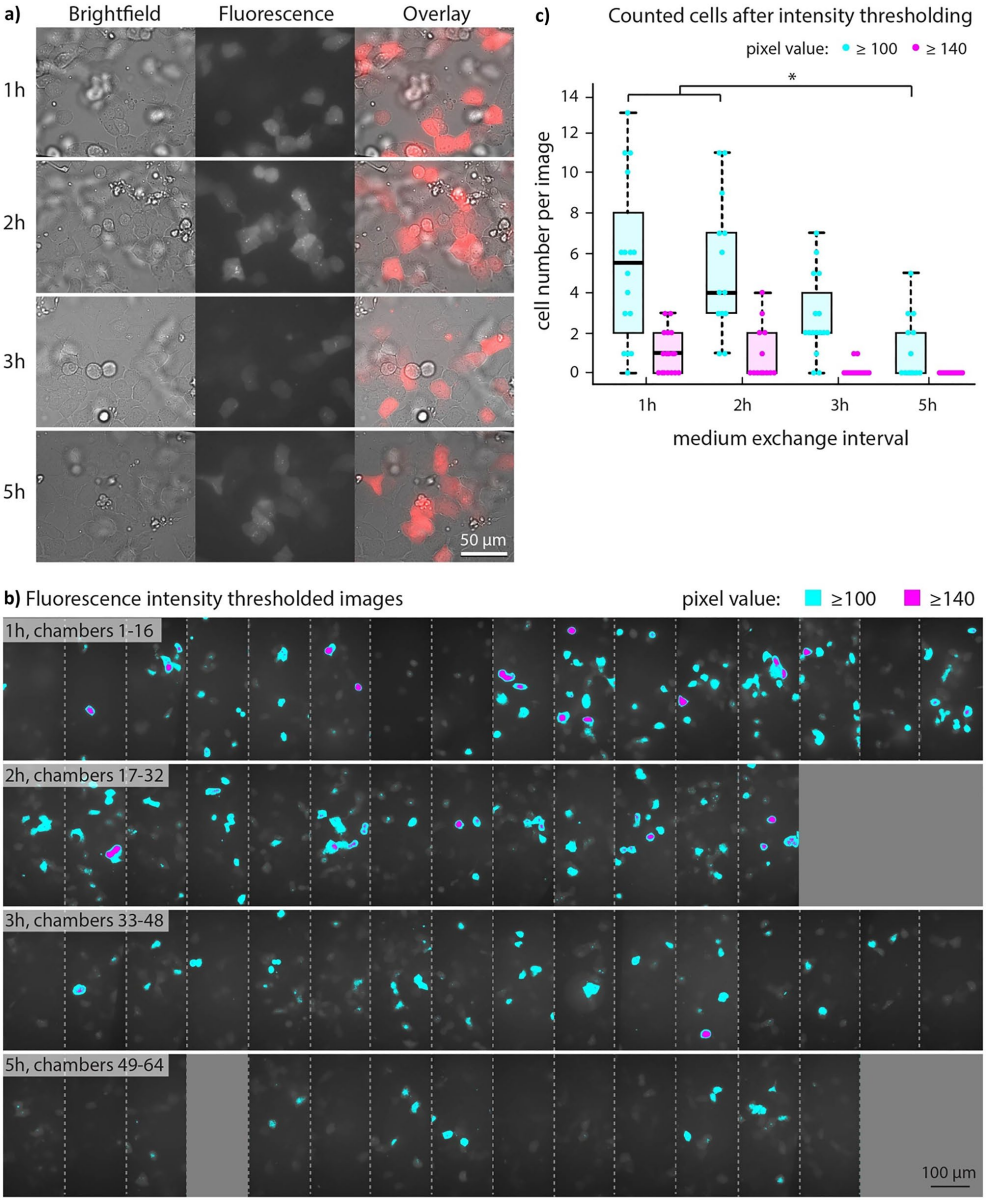
MESP1^mCherry^ expression on day 3 of hESC differentiation towards cardiac mesoderm. The chambers with shorter medium exchange intervals contain more cells which have brighter fluorescence. (**a**) Brightfield (left), fluorescence (center), and overlaid (right) images of the cells on day 3 in chambers with 1 h, 2 h, 3 h, and 5 h medium exchange intervals. (**b**) MESP1^mCherry^ fluorescence images of chambers with 1 h, 2 h, 3 h and 5 h medium exchange intervals. The blue areas represent pixel values greater than 100 and the magenta areas represent pixel values greater than 140. Each picture was taken in a different chamber. No images are available for the six chambers represented by grey rectangles. (**c**) Boxplot of the number of cells per chamber which have a fluorescence intensity corresponding to pixel values above the thresholds 100 (blue) and 140 (magenta). A Kruskal–Wallis test with Dunn’s pairwise comparisons showed a significant difference (*p* < 0.05) between the 1 h and 5 h as well as the 2 h and 5 h intervals for the 100 threshold. No test was performed on the 140 threshold due to the small number of cells above this threshold

MCherry fluorescence was observed using microscopy on day 3 in all of the chambers containing cells, regardless of whether these had 1 h, 2 h, 3 h, or 5 h medium exchange intervals. Figure 5a shows an example image of a chamber from each condition. The fluorescence and brightfield overlay images show that several, but not all cells in each chamber express MESP1. Similar results are also observed in the original well plate protocol (see Supplementary Fig. 2b for comparison).

MESP1^mCherry^ fluorescence is often particularly bright in the 1 h and 2 h chambers. Figure 5b shows an array of images, whereby each image was taken in a different chamber and each row corresponds to a different medium exchange interval. The cells with the highest fluorescence intensity are shown colored, whereby blue and magenta correspond to a pixel value ≥ 100 and ≥ 140, respectively. The thresholds were chosen at about 1.25 and 1.75 times the background noise (values around 80) which overlaps with the fluorescence signal from mCherry in several images. The number of cells per image with intensities above these thresholds were counted and are shown in boxplots in Fig. 5c. The blue boxplots (≥ 100 threshold) show a wide spread for all conditions. The interquartile range for the 1 h and 2 h boxes encompass approximately the same values. For the 3 h and 5 h intervals a clear trend toward decreasing cell numbers is observed. A Kruskal–Wallis Test was performed on the 100 threshold and showed a significant difference between the mean ranks (*p* = 0.001) of at least one pair of the datasets. Dunn’s pairwise tests revealed a significant difference between the 1 h and 5 h intervals (*p* = 0.003) as well as between the 2 h and 5 h intervals (*p* = 0.002), after the Bonferroni correction for multiple comparisons. Due to the low number of cells above the 140 threshold, the test was not performed on this threshold.

The decrease in fluorescence intensity for longer medium exchange intervals (5 h), may be due to a delayed peak MESP1 expression compared to the 1 h and 2 h chambers. In another experiment MESP1^mCherry^ fluorescence was imaged on day 2 of differentiation for 1 h and 2 h medium exchange intervals. Both conditions showed MESP1 expression, demonstrating that at least for these two conditions the cells already enter the cardiac mesoderm stage on day 2. To confirm whether the fluorescence intensity difference is due to differentiation with a higher efficiency or to a time-shifted differentiation, further experiments with live-cell imaging of MESP1^mCherry^ have to be performed.

## Conclusion and outlook

In this article we showed that we can differentiate hESCs to cardiac mesoderm cells in 30 nL microchambers using different discontinuous medium perfusion intervals. We can parallelize up to 64 of these chambers on an automated mLSI chip. Since all chambers can be addressed with spatiotemporal independence, differentiation medium can be delivered to the cells at different time intervals. We tested intervals between 1 and 5 h and showed that MESP1-positive cells could be produced by day 3 in all cases. However, the cell self-reorganization on days 1 and 2 as well as the brightness of MESP1^mCherry^ on day 3 varied with different medium exchange intervals. Shorter exchange intervals (1 and 2 h) led to compacted aggregation of the cells on day 1 and cell dispersion from these aggregates by day 2. The cells in these chambers generally also showed brighter MESP1^mCherry^ fluorescence on day 3 compared to cells in chambers treated with longer exchange intervals (5 h). Moreover, the differentiation in the chambers led to a twice as high yield in cells with MESP1 expression compared to the differentiation in the well plate. These results underline the importance of tightly controlling the cells’ microenvironments to improve cell lineage commitment and show that differentiation in nanoliter volumes are a promising way to achieve this.

Having validated our chip for the directed differentiation of hESCs, we plan to use our previously introduced fluidic circuit board system (Vollertsen et al. 2020) to parallelize three of our 64-chamber chips. Using the upscaled system to test more conditions, we plan to extend the protocol to full cardiomyocyte differentiation. The cell line that was used in this article, MESP1^mCherry/w^-NKX2.5^eGFP/w^ hESCs (Den Hartogh et al. 2015) also contains a reporter for NKX2.5 which becomes visible around day 8. This reporter can be used as an intermediate read-out for the next stage of differentiation. Finally we hope to be able to obtain beating cardiomyocytes in the chambers around day 13.

In the future, further optimization of the differentiation protocol may be achieved by also varying the concentration of growth factors in the cell medium. This way more ratios of cell-secreted to externally provided factors can be tested. Specifically, higher concentrations of exogenous factors may be used to lengthen the time between medium exchanges, giving the cell-secreted factors more time to accumulate. Thereby, enhancing or inhibitory effects at different levels of cell-secreted factors might be elucidated. Moreover, the dynamic stem cell microenvironment can be further simulated by changing this ratio according to the stage in differentiation. Due to the small volume of the chambers, high temporal resolution of modulating the microenvironment can be achieved without compromising the accumulation of cell-secreted factors.

## Materials and methods

### Chip fabrication

The 64-chamber chip was designed and fabricated as reported by Vollertsen et al. (2020). Briefly, the photolithography mask was designed in Clewin Layout Editor (Wieweb software) and used to fabricate two silicon wafer molds (one for the flow layer and one for the control layer) by standard photolithography. All structures with a rectangular profile on either wafer were made with SU8 photoresist. In places where there are valves on the flow layer, AZ40XT photoresist was used to create channels with a rounded profile. The PDMS (RTV615, Permacol, The Netherlands) chips were fabricated from these molds by standard multilayer soft lithography. To bond the flow and control PDMS layers different ratios of PDMS base polymer to curing agent were used for the flow layer (7:1, base polymer to curing agent) and control layer (20:1). The control layer thickness of approximately 40 µm was achieved by spin-coating the PDMS on the wafer. Both layers were cured at 60 °C for 45 min. Subsequently, the flow layer in- and outlets were punched using a 1.0 mm hole puncher. Next, the flow layer was aligned on top of the control layer using an Olympus stereo microscope and both layers were cured overnight (at 60 °C). The control layer holes were punched using a 0.75 mm hole puncher. Finally, the PDMS layers were oxygen plasma-bonded to a 1 mm thick microscope glass slide. For MESP1 imaging, an oil objective with a working distance of 0.2 mm was used. Therefore, chips for these experiments were bonded to a 0.2 mm thick cover glass slide instead of a microscope slide.

### Automation set-up

The valves in the control layer are actuated mechanically by air pressure. The control channels of the chip were connected to solenoid valves which switch between high (1.7 bar) and atmospheric pressure, whereby compressed air is used as a high pressure source. A pressure pump (Flow EZ™, Fluigent, Germany) regulated the flow from a fluid reservoir to the flow layer. Tygon® tubing with an inner diameter of 0.5 mm connected the inlets of the chip to the external components. A custom LabView program controlled both the solenoid valves and the pressure pump.

### Microscope incubation system

The microscope incubation system was the same one as was previously reported by (Vollertsen et al. 2020). Briefly, a heating plate insert on a microscope stage maintained the chip’s temperature at 37 °C. The heating plate temperature could be adjusted using the aforementioned custom LabView program. A 10 kΩ Vishay temperature sensor recorded the temperature via a NI MyDAQ system and the custom LabView program. A CO_2_ controller (Okolab, Italy) delivered air with 5% CO_2_ into a custom built box on the microscope stage. The 5% CO_2_ air was bubbled through a water bath located inside the box to humidify the air. Additional wells filled with water were placed next to the chip to increase the humidity and thereby prevent the chip from drying out. The removable box lid allowed easy access to the chip when connecting tubing and seeding cells. The tubing from the chips was connected to the external components via a box insert which could be exchanged to accommodate for different sizes or amounts of tubing. To keep the chip in the dark, blackout cloth was draped over the microscope.

### HESC culture and differentiation protocol in well plates

A previously generated MESP1^mCherry/w^-NKX2.5^eGFP/w^ human embryonic stem cell (hESC) dual reporter line (Den Hartogh et al. 2015) was maintained in standard wells plates coated with vitronectin (Thermo Fisher) and cultured in Essential 8 (E8) medium (Thermo Fisher) supplemented with 1% penicillin/streptomycin (Thermo Fisher). HESCs were passaged twice a week using EDTA (Thermo fisher).

**Fig. 6:**
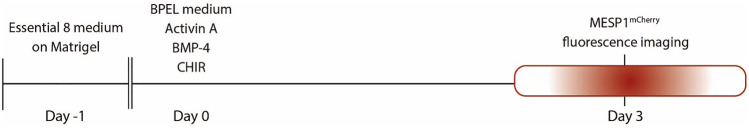
A schematic overview of the differentiation protocol towards mesodermal lineage. MESP1^mCherry^ expression is indicated in red around day 3

Control experiments for cell differentiation were performed in 6-well plates in parallel to on-chip differentiation. On day -1, hESCs were seeded at 125 × 10^3^ cells/mL in E8 medium supplemented with 1% penicillin/streptomycin on a Matrigel-GFR (VWR) coated 6-well plate. On day 0, the cells were washed with in-house made BPEL (BSA, Polyvinyl alcohol, Essential Lipids) medium (Ng et al. 2008), before adding growth factor-supplemented medium (20 ng/mL Activin A (Miltenyi Biotech), 20 ng/mL BMP-4 (R&D Systems), and 1.5 µM CHIR-99021 (Sigma Aldrich)) in BPEL medium at 3 mL per well. The differentiation timeline is shown in Fig. 6. All cell cultures were maintained at 5% CO_2_, 37 °C, and atmospheric O_2_ levels.

### MFBB surface functionalization and hESC loading

The channel (chamber) surfaces were functionalized to prevent (promote) cell adhesion. To achieve this, the chip was first exposed to oxygen plasma to create silanol groups on the PDMS surface. Next, the chip was connected to the automation system and the control channels were filled with sterile-filtered DI water. The air in the dead-end control channels was pushed out through the PDMS when the control channels were pressurized. Subsequently, the flow channels were filled with autoclaved and sterile-filtered DI water to stabilize the silanol groups (Makamba et al. 2003). All chambers were closed off and 100 µg/mL PLL-g-PEG (poly(L-lysine) poly(ethylene glycol)) (SuSoS, Switzerland) in phosphate-buffered saline (PBS) solution was flushed through all channels for approximately one hour. Next, the channels were purged of the PLL-PEG solution and filled with a 100 µg/mL Matrigel solution. The chambers were opened and flushed with the same Matrigel solution for 3 min. The heating stage was then set to 37 °C and the chip was incubated for approximately one hour. Adding water on top of the chip prevented the flow layer from drying out and hence bubbles from forming. Prior to cell seeding, the chip was flushed with E8 medium. HESCs were seeded through a pressurized pipette tip (as described by Rho et al. (2015)) at 4 · 10^6^ cells mL^−1^ and 50 mbar. The chambers were filled sequentially with two rounds of seeding in chambers 1–32 followed by two rounds of seeding in chambers 33–64. Each round took about 3 min which was sufficient time for the hESCs in the first chambers to attach to the chamber floors before the next seeding round started. The cells were incubated for 1 h before the automated medium refreshment protocol started.

### Image analysis for hESC counting during proliferation

The number of cells per chamber was determined by analyzing the brightfield images using a custom ImageJ (version 1.52p) macro. This macro executed the following steps: First, the RGB image was spilt into color channels and only the red channel was used for further processing. Next, a FFT bandpass filter was applied to filter out the background (structures down to 10 pixels) and despeckle the image (filter structures up to 4 pixels). The default tolerance of direction (5%) was used. Since the image histograms lacked two clearly distinguishable peaks, a local threshold was applied (method Bernsen with a radius of 120 pixels and default (= 0) parameters 1 and 2). The resulting was inverted and the process ‘watershed’ was applied to split overlapping ellipsoids. Finally, the number of particles was counted for each chamber (using ‘analyze particles’), whereby only particles with an area of 25–300 pixels and a circularity of 0.30 – 1.00 were considered. For each set of images of the 64 chambers, cells in 8 chambers were counted and marked manually. The images of the manual counts and the particle masks resulting from the macro were overlaid and compared. If necessary, the particle area and circularity were adjusted.

In images taken an hour after seeding, the cells had a different contrast and therefore the local threshold method ‘Phansalkar’ (radius of 120 pixels and default (= 0) parameters 1 and 2) provided better results. The macro cell count in these images was also often distorted by debris in the chambers. To solve this, the blue channel image was processed to detect debris by subtracting the background (rolling = 50, light, sliding) and applying the local threshold (‘Phansalkar’, radius of 50 pixels and default (= 0) parameters 1 and 2). The resulting image was inverted and a particle mask was created (‘Analyze particles’, an area of 4–200 pixels and a circularity of 0.3–1.0). The debris mask was then subtracted from the thresholded red channel image. The remaining particles (cells without debris) were counted in the final step and compared with manually counted images.

### Statistical analysis

The data subset shown in Fig. 2b shows chambers which are filled with approximately the same number of cells 1 h after seeding. The subset of chambers were selected blindly in regard to the 16 h and 24 h values. A mixed design repeated measures ANOVA with three levels (one for each time point) was performed on the cell numbers per chambers in each condition using OriginPro 2019b. Mauchly’s Test of Sphericity showed a violation of sphericity (*p* < 0.001), with the Greenhouse–Geisser and Huynh–Feldt Epsilons equal to 0.639 and 0.712, respectively. Since the Greenhouse–Geisser Epsilon was smaller than 0.75, this correction was applied. With this correction, the Tests of Within-Subjects Effects showed no significant interaction between the culturing time and the interval with *p* = 0.055. A mixed design repeated measures ANOVA with two levels was performed on the cell population increase percentages (shown in Suppl. Fig. 1). For the experiment shown in Supplementary Fig. 2 a mixed design repeated measures ANOVA was performed on the cell numbers per chamber and time point. A one way ANOVA was also performed on the cell population increase percentages.

The Kruskal–Wallis test and Dunn’s pairwise comparisons with Bonferroni adjustments on the cell numbers in Fig. 5c were performed using IBM SPSS® version 26.

### HESC fixation and staining

HESCs were fixed for 30 min with 4% v/v paraformaldehyde in PBS by flushing each chamber for 5 s to 10 s in several rounds. Afterwards, the paraformaldehyde solution was purged with PBS. A 0.1% Triton-X + 1% BSA solution was used to permeabilize the cells for 1 h at room temperature. Cell staining in the well plates was performed with a DPBS solution containing DAPI and Alexa Flour®-conjugated antibodies for OCT3/4 (2 µg/mL, Santa Cruz Biotechnology) and SOX2 (5 µg/mL, Thermo Fisher) and 1% BSA (Bovostar). NucBlue (Thermo Fisher) and a 20 × higher concentration of the same Alexa Flour®-conjugated antibodies were used to stain the cells in the chambers. The chip was filled with this solution and subsequently disconnected from the automation set-up. A pipette tip filled with the staining solution was inserted into one inlet and an empty pipette tip into the outlet. All other inlets were plugged using stainless steel 1 mm diameter pins. The chip was stored at 4 °C overnight. hESCs in the control wells were also fixed with 4% v/v paraformaldehyde in PBS and permeabilized with Triton-X + 1% BSA solution for 1 h at room temperature. 200 µL of the same antibody solution were added per well in a 12-wells plate and was stored at 4 °C overnight. The cells were imaged with an EVOS FL2 microscope using DAPI, GFP (for SOX2) and Cy5 (for Oct 3/4) filter cubes for the fluorescent images.

For cells used as a negative control in the pluripotency immunostaining, the cells on day 3 were fixated with a 4% formaldehyde solution.

### HESC differentiation in chip

For the first 24 h after hESC seeding (day -1 to day 0), the E8 medium in the chambers was fully exchanged in programmatically set intervals. To test the effect of different medium exchange intervals on hESC pluripotency and proliferation, intervals between 1 and 8 h were chosen for different chambers. For cardiac mesoderm differentiation experiments, the exchange interval was set to 2 h for all chambers. On day 0, the cells were either fixed (for pluripotency staining) or the chambers were all flushed with BPEL medium and subsequently growth factor-supplemented (20 ng/mL Activin A (Miltenyi Biotech), 20 ng/mL BMP-4 (R&D Systems), and 1.5 µM CHIR-99021 (Sigma Aldrich)) BPEL medium. The program was reset to exchange the medium at new intervals (between 1 and 5 h). Every medium exchange had a duration of 7 s per chamber and the pressure for the flow was set between 35 and 40 mbar.

### Quantification of MESP1 expressing cells on day 3 of differentiation

The cells cultured in the chambers had medium refreshment intervals of 2 h on day 0, 3 h on day 1, and 4 h on day 2. All chambers had the same conditions. The cells were dissociated in both the well plate and the chip on day 3 of the differentiation using 1 × Tryple (Thermofisher). After centrifugation, the cells were resuspended in PBS supplemented with 0.5% BSA (Catusbiotech) and 2 mM EDTA (Thermofisher). Fluorescence measurements for mCherry were accomplished by flow cytometry (MACSQuantify, VYB). MESP1^mCherry/w^-NKX2.5^eGFP/w^ hESCs were used as a negative control.

### MESP1 live cell imaging

On day 3 of differentiation the chip was disconnected from the automation set-up and moved to a Nikon EclipseTE2000-U fluorescence microscope for live-cell imaging. The MESP1^mCherry^ reporter was visualized using a Nikon CFI Super Fluor 40 × oil immersion objective (MRF01400), a RFP filter cube and a Basler ace camera (model acA4096-40um, Basler AG, Germany). Control cells in well plates were imaged likewise. The fluorescence images which were taken are 8-bit images with 256 Gy levels. The image thresholding was performed using ImageJ (version 1.52p). The images were stitched using IrfanView (version 4.56) and the boxplot was made using OriginPro 2019b.

## Supplementary Information

Below is the link to the electronic supplementary material.

Supplementary file1 (DOCX 1590 KB)

Supplementary file2 (GIF 1460 KB)
