# Supplementary material for: Highly parallelized human embryonic stem cell differentiation to cardiac mesoderm in nanoliter chambers on a microfluidic chip
Source: Biomed Microdevices. 2021 May 31;23(2):30. doi: 10.1007/s10544-021-00556-1 (PMC8166733; doi:10.1007/s10544-021-00556-1)
Supplement: Supplementary file 1 — Supplementary file1 (DOCX 1590 KB) [file 10544_2021_556_MOESM1_ESM.docx]

**Supplementary Information**

**S1 Statistical analysis on HESC proliferation with E8 medium exchange intervals**

Supplementary Figure 1a shows the percentages by which the population increased (on average) in each chamber per condition. These percentages are between 26% (8h) and 54% (3h) after 16h and between 47% (8h) and 85% (3h) after 24h of culturing. Tests of Within-Subjects Effects for an interaction between the culturing time and the medium exchange yielded p = 0.061.

Although both Figures 2b and Supplementary Figure 1a seem to show lower cell proliferation in the 8h chambers, no statistically significant difference between the conditions at each time point was found after a repeated measures ANOVA (analysis of variance). Tests of Within-Subjects Effects for an interaction between the culturing time and the medium exchange showed no significant effect with p = 0.37.

The p-value which gives an indication that the medium exchange interval has an effect on the cell proliferation is p = 0.055 and therefore may still show statistical significance (p < 0.050) in experiments with more chambers per condition. To more robustly define a range in which the medium exchange interval does not negatively impact the cell proliferation (within 24h), another experiment with 1h, 2h, 3h, and 5h medium exchange intervals was performed. Supplementary Figure 1b shows the cell population in each chamber (black triangle) for every medium exchange interval 2h and 21h after seeding. The ANOVA shows no statistically significant effect of the different intervals on the cell proliferation after 21h of culturing (p = 0.37). After 21h, the mean cell population per condition had increased by approximately the same percentage (no significance was determined by a one-way ANOVA, p = 0.98).


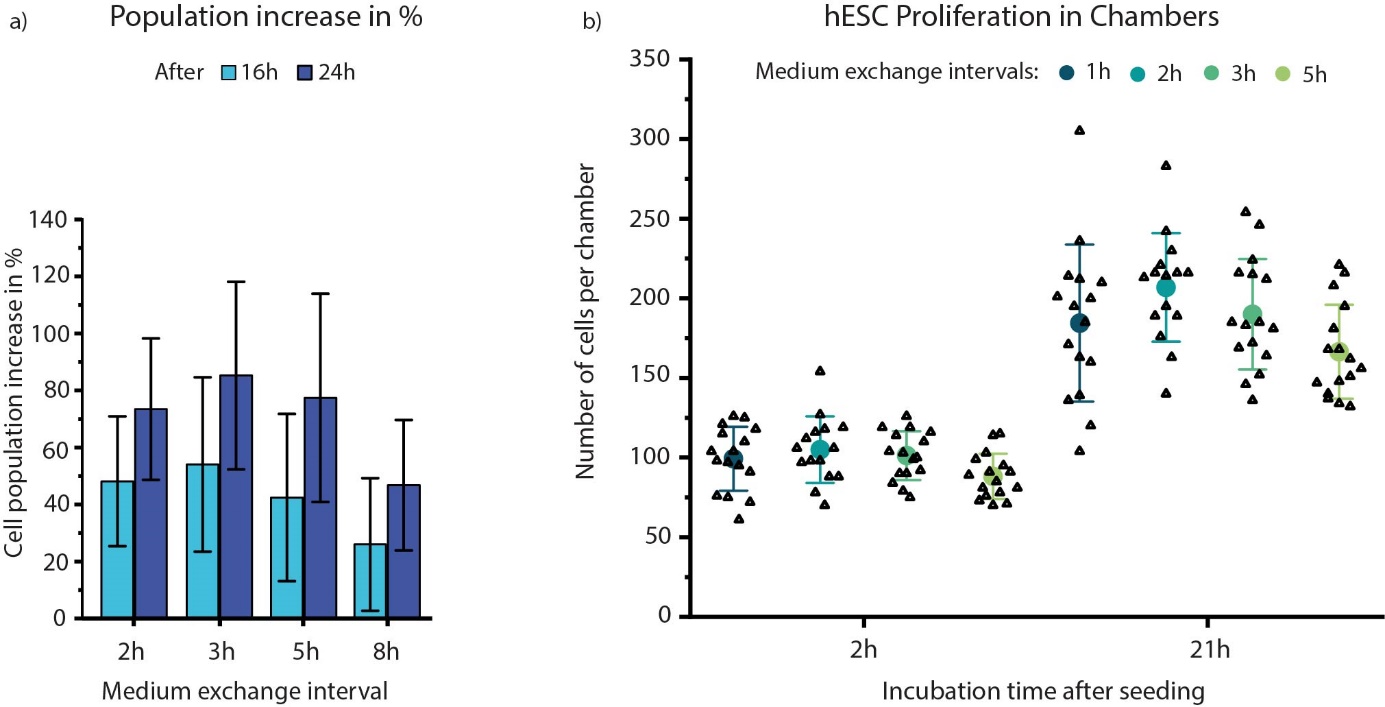


Supplementary Figure 1: a) Percentual increase in cell population 16h and 24h after cell seeding for the same chambers as in Figure 2b). The error bars represent the standard deviation. No significant difference was found between the conditions per time point using a repeated measures ANOVA. b) HESC populations in microfluidic chambers with 1h, 2h, 3h, and 5h medium exchange intervals. The mean number of cells per chamber is represented by the circles. The error bars represent the standard deviations.

**S2 HESC differentiation in well plates**

Controls for the hESC differentiation in the chambers was performed in well plates. Cell aggregation and dispersion along with morphology change is shown in Suppl. Figure 2a. The confirmation of early cardiac mesoderm cells by MESP1^mCherry^ expression on day 3 is shown in Suppl. Figure 2b.


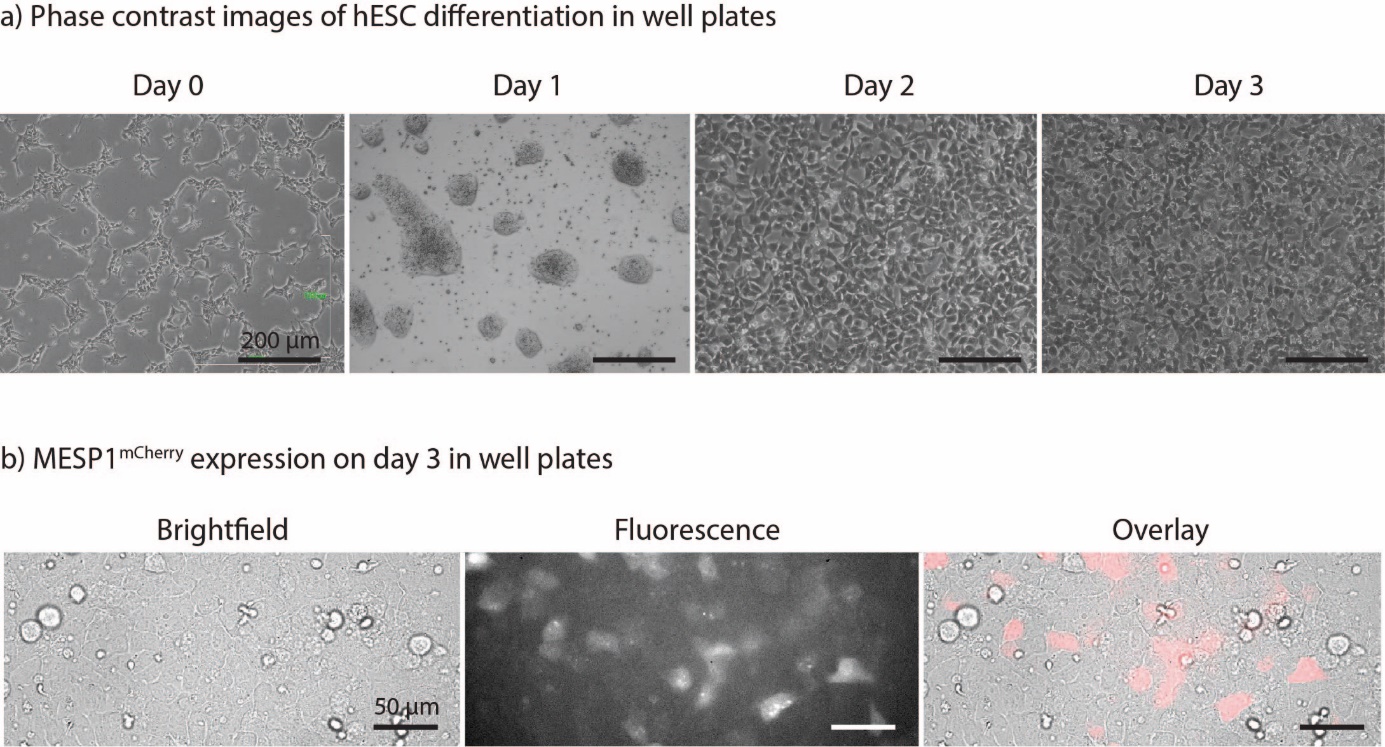


Supplementary Figure 2: HESC differentiation in well plates. a) Phase contrast images of the differentiation from pluripotent (day 0) to cardiac mesoderm (day 3). b) MESP1^mCherry^ expression on day3 shown by fluorescence live-cell imaging.

**S3 High cell number at start of differentiation**

Suppl. Figure 3 shows an experiment with 1h, 2h, 3h, and 5h intervals for differentiation medium exchange. In comparison to Figure 3, differentiation was started with approximately twice as many cells per chamber. Although the same trends can be observed on day 1 in both cases (namely the compacting of the cells at shorter medium exchange intervals), there is no dispersion of the aggregates on day 2. Furthermore, the chambers are overfilled with cells and cell aggregates on day 3 in all conditions.


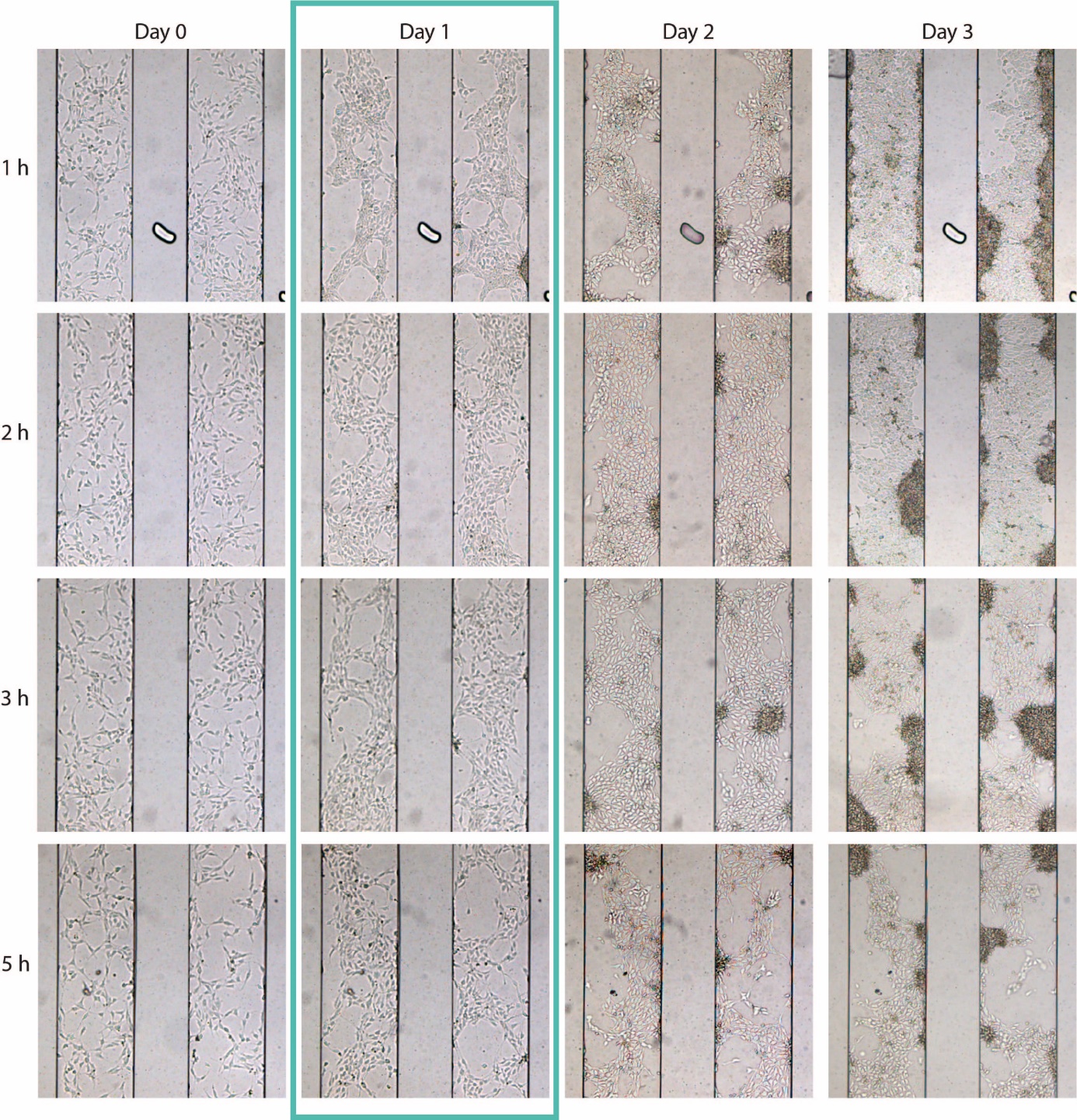


Supplementary Figure 3: HESC morphology and re-organization during differentiation with higher cell densities at day 0 (300-400 cells per chamber). By day 3, the chambers are overfilled with cells.

**S4 Flow cytometry**

Supplementary Figures 4 a) and b) show the flow cytometry data in histograms and density plots respectively. hESCs were used as the negative control and cardiac mesodermal cells from both the well plate and the chip chambers were measured to determine the percentage of the population which expressed MESP1.


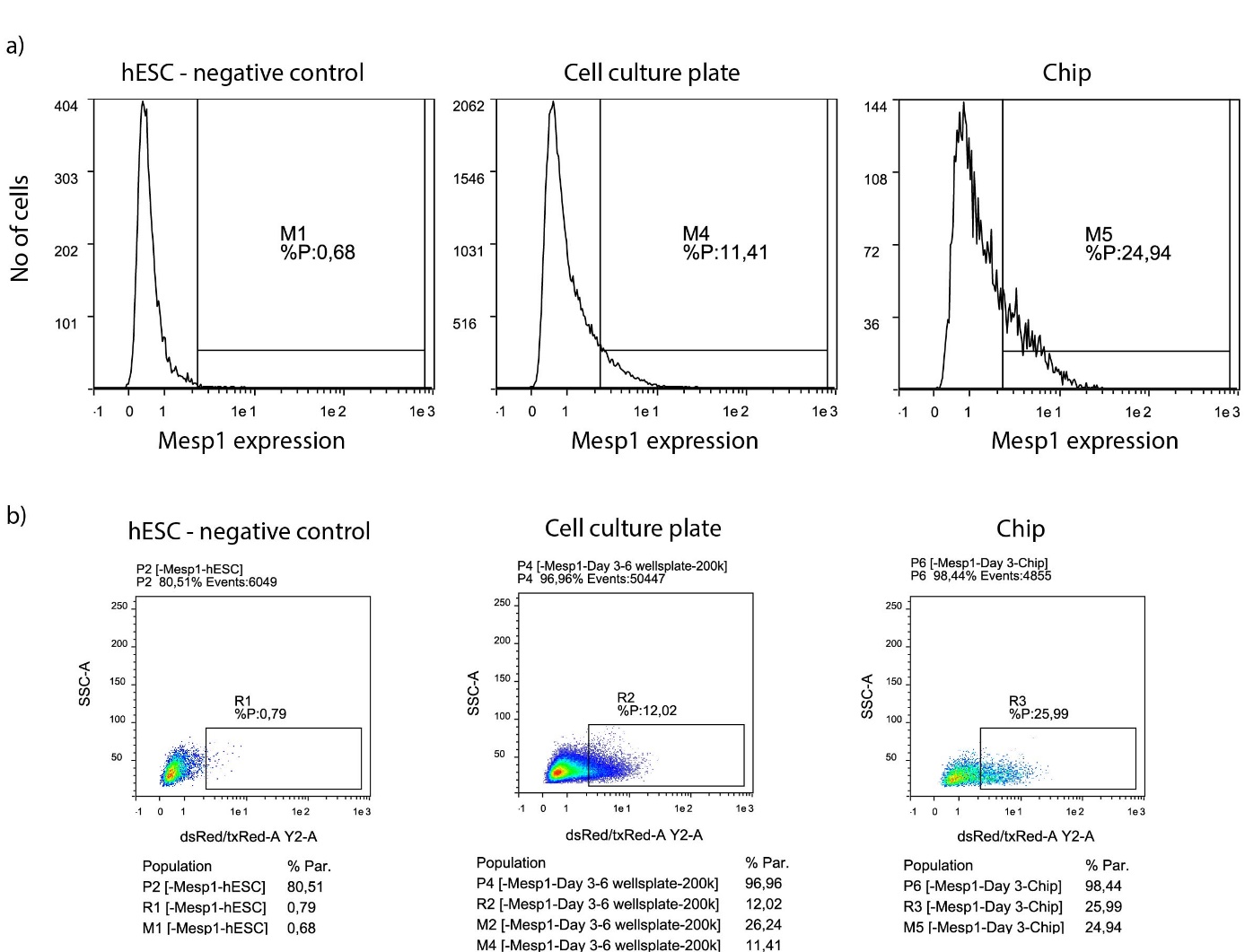


Supplementary Figure 4: Flow cytometry data as a) histograms and b) density plots.

**S5 Fluorescence image histograms**

Supplementary Figure 5 shows histograms of the fluorescence images shown in Figure 5. Supplementary Figure 5a shows two representative histograms of two images: one of chamber 22 (red) and one of chamber 44 (orange). The black dashed line shows were the approximate cutoff for the background noise was determined. The blue and magenta dashed lines indicate the chosen thresholds for Figure 5 at 1.25 and 1.75 times the background noise cutoff. Supplementary Figure 5b shows an averaged histogram created from all the fluorescence images shown in Figure 5 for each condition. The histograms (especially for the 2h) show a shoulder between pixel values of 100 and 150, indicating an underlying species.


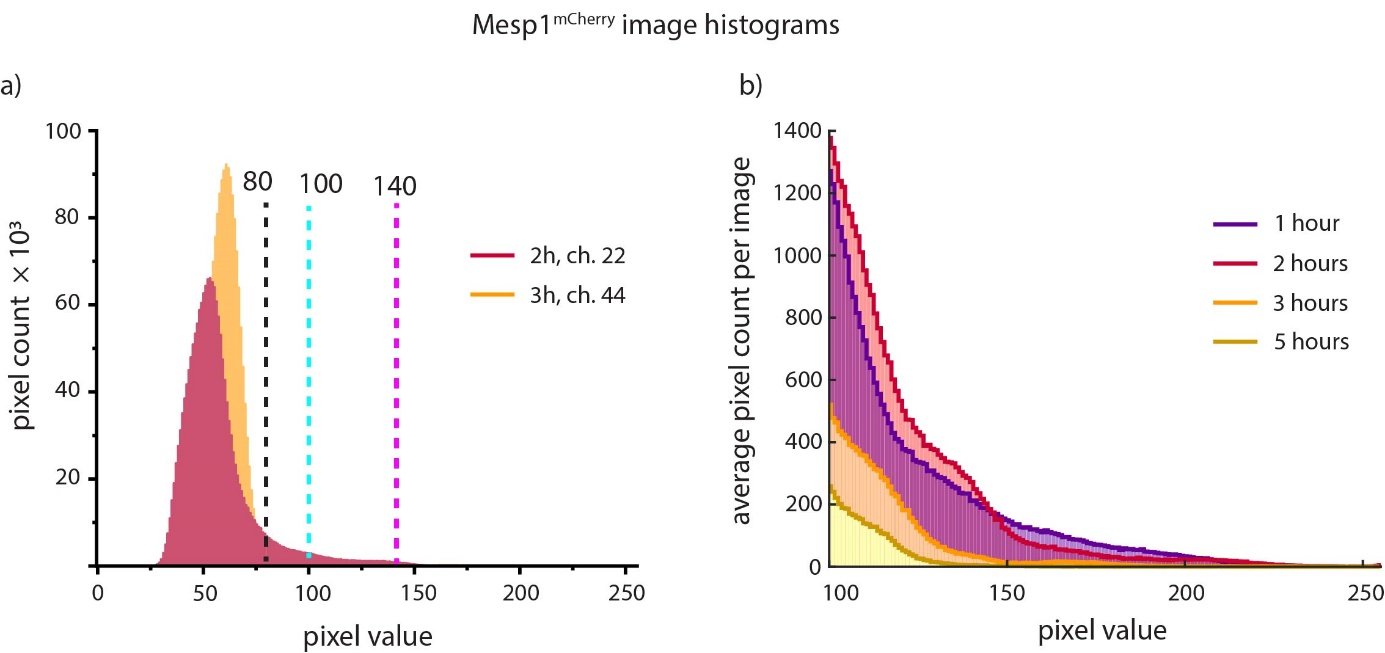


*Supplementary Figure 5: Histograms of the fluorescence images shown in Figure 5. a) Two representative full histograms. b) Close-up of the pixel value range 100-255 for averaged histograms created for each condition.*
