# Supplementary figures and images for: Highly parallelized human embryonic stem cell differentiation to cardiac mesoderm in nanoliter chambers on a microfluidic chip
Source: Biomed Microdevices. 2021 May 31;23(2):30. doi: 10.1007/s10544-021-00556-1 (PMC8166733; doi:10.1007/s10544-021-00556-1)

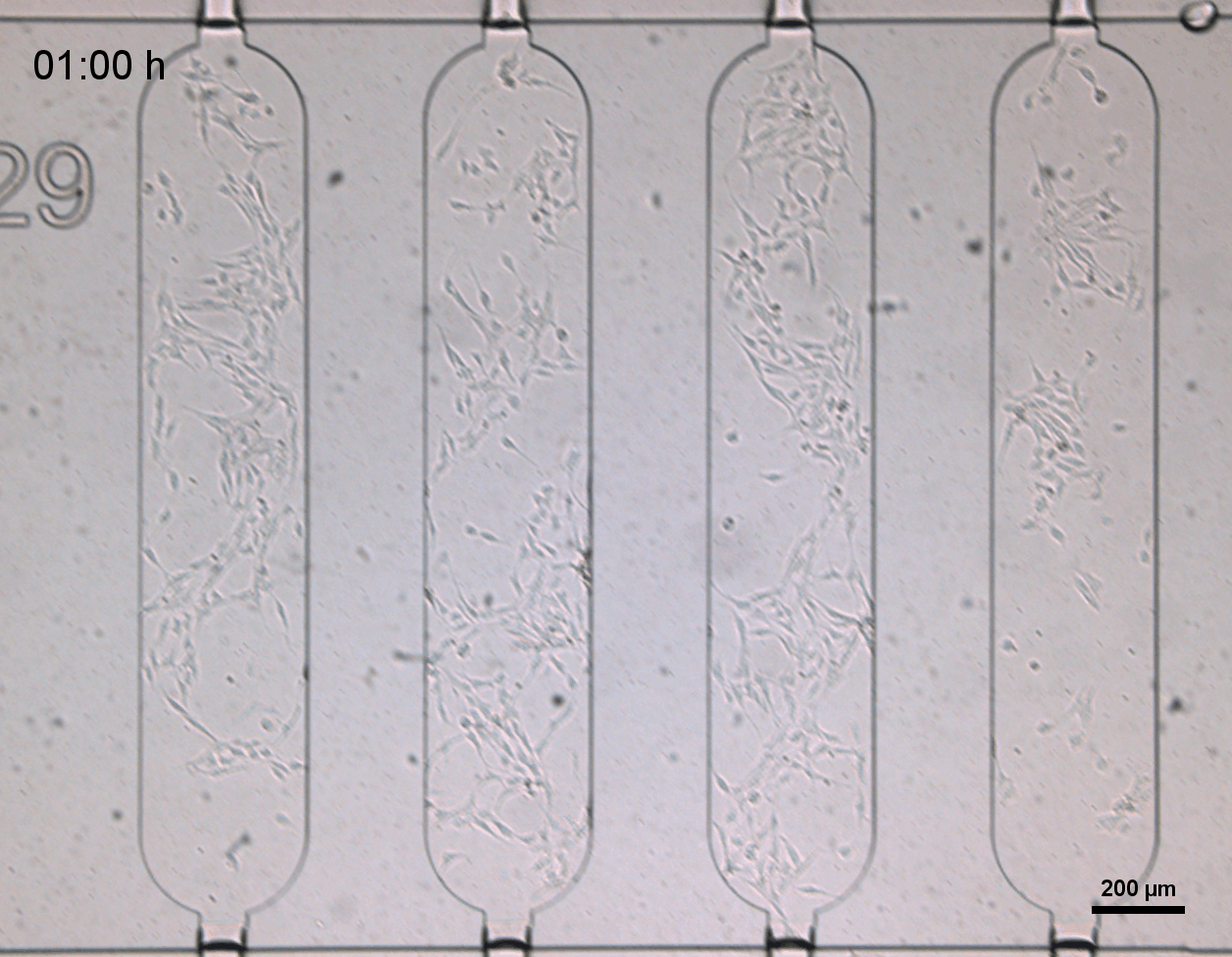

Supplement: Supplementary file 2 — Supplementary file2 (GIF 1460 KB) [file 10544_2021_556_MOESM2_ESM.gif]
